# Skinks on a Plane: Does Human‐Mediated Transportation Impact the Behaviour of an Invasive Lizard?

**DOI:** 10.1002/ece3.70748

**Published:** 2024-12-18

**Authors:** Jaclyn Harris, Celine T. Goulet, David G. Chapple

**Affiliations:** ^1^ School of Biological Sciences Monash University Clayton Victoria Australia

**Keywords:** activity, biological invasion, exploratory behaviour, human‐mediated dispersal, *Lampropholis delicata*, squamate

## Abstract

The human‐mediated transportation of stowaway individuals to non‐native regions is a major driver of new biological invasions, and the post‐establishment spread of the invader in its introduced range. In order for the stowaway individuals to successfully establish in the non‐native region, they must survive the harsh conditions during the journey (e.g., extreme temperatures, cramped spaces, and lack of food) and arrive in good condition. However, few studies have investigated the impact of human‐assisted transportation on the behaviour of stowaway individuals. Here, we examined whether human‐mediated transportation, via both air and road, impacted the activity and exploratory behaviour of the invasive delicate skink ( *Lampropholis delicata* ). We exposed delicate skinks to either flights on a commercial airliner (total ~2.5 h flight time, and car transport to/from the airport), or a 3 h drive in a car. We found that although the temperatures experienced by skinks during transportation were more variable than those experienced by control group lizards, the temperature during transit remained well within the thermal tolerances for the species. Human‐assisted transportation only had a relatively minor impact on the behaviour of the invasive delicate skink: transportation by plane did not influence activity or exploratory behaviour, and car transportation increased activity, but did not impact exploratory behaviour. The capacity of stowaways to cope with the stress associated with human‐mediated transportation is a key factor in the success of species introductions, and subsequent invasion. As such, there should be a greater focus on the behaviours that facilitate the success of potential invaders in the early stages of the introduction process.

## Graphical abstract

In order for invasive species to successfully establish in the non‐native region, they must survive the journey and arrive in good condition; however, few studies have investigated the impact of human‐assisted transportation on the behaviour of stowaway individuals. In our study, we exposed lizards to air and road transportation to investigate the impact of this transportation on the subsequent behaviour of individuals. We found that human‐assisted dispersal only had a relatively minor impact on the behaviour of the invasive delicate skink. We conclude that the capacity of stowaways to cope with the stress associated with human‐mediated transportation is a key factor in the success of species introductions, and subsequent invasion.

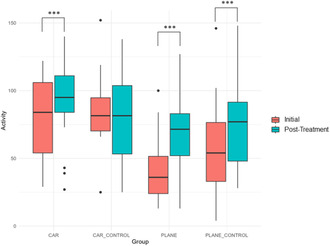

## 1 Introduction

Invasive species are a major threat to biodiversity, and have been a leading cause of native species extinctions (Doherty et al. 2016; IPBES 2023). Increasing globalisation has led to a surge in stowaway animals being transported to areas outside of their native range through human‐mediated dispersal (White and Shine 2009; Hulme 2009; IPBES 2023). For these unintentional introductions to result in successful biological invasions, the stowaways must successfully navigate their way through each sequential stage of the introduction process: transport, introduction, establishment and spread (Blackburn et al. 2011; Chapple, Simmonds, and Wong 2012). Despite the relatively low, but variable, success rate of unintentional species introductions at each stage of the invasion process (Williamson and Fitter 1996), unintentional human‐mediated dispersal is the mechanism through which numerous species spread and become invasive (Suarez, Holway, and Case 2001; Wilson 2009; Chapple, Simmonds, and Wong 2012; Gippet et al. 2019).

In order for stowaways to successfully establish in a non‐native region, they must survive the journey in the transport vector (e.g., plane, cargo ship, truck), and arrive at the new location in good health and condition (Chapple, Simmonds, and Wong 2012; Chapple and Wong 2016). However, the conditions that the stowaways experience during transit may be harsh, and variable; for example, low oxygen, limited space, extreme temperatures, an extended journey, loud/novel sounds, and a lack of food and/or other key resources (Ruiz and Carlton 2003; Hulme et al. 2008; Hulme 2009). Whilst some biosecurity agencies record the mortality rate of stowaway animals (e.g., New Zealand; Chapple et al. 2016), the sublethal impacts of transportation on stowaways has rarely been determined (but see Mancera et al. 2014; Petit, Greenlees, and Shine 2017a, 2017b). This is surprising, as presumably species that are more tolerant of the conditions experienced during transit may have a higher likelihood of arriving in good condition, and ultimately have increased chances of successful establishment in the non‐native region. Given this, we aimed to determine whether human‐assisted transportation impacts the behaviour of a well‐characterised, prolific invader, the delicate skink (*Lamproholis delicata*).

The delicate skink is native to eastern Australia (Chapple et al. 2011), but has successfully established, and subsequently become invasive, in the Hawaiian Islands, New Zealand, and Lord Howe Island [~750 km NE of Sydney] (Chapple, Miller, et al. 2013; Chapple, Miller, Chaplin, et al. 2014; Chapple, Reardon, and Peace 2016; Tingley et al. 2016). The delicate skink is highly adept at human‐assisted dispersal, and is a more frequent stowaway in freight, cargo and personal baggage than closely related congeners (i.e., the garden skink, *Lampropholis guichenoti*), with equivalent opportunity for transportation (Chapple, Simmonds, and Wong 2011; Chapple, Whitaker, et al. 2013; Chapple, Knegtmans, et al. 2016; Cromie and Chapple 2012; Pili et al. 2023). For instance, the delicate skink is the skink species that is mostly commonly intercepted by biosecurity agencies entering New Zealand (Chapple et al. 2016), and has been recorded as a stowaway in air, sea, and road/rail transport vectors (Chapple, Miller, et al. 2013).

Here, we examine whether human‐assisted dispersal, via both air and road, impact the behaviour of the delicate skink. Both activity and exploratory behaviour are associated with invasion success in a range of species (Chapple, Simmonds, and Wong 2012), and have been demonstrated to be under selection during the introduction process in the delicate skink (Chapple et al. 2022). Importantly, these behaviours are repeatable in the delicate skink (Michelangeli, Wong, and Chapple 2016; Polverino et al. 2023), and exhibit few differences between the sexes (Michelangeli, Chapple, and Wong 2016). Although previous studies have either simulated transport conditions (e.g., Mancera et al. 2014) or focused on a single transport method (e.g., road vehicle; Petit, Greenlees, and Shine 2017a, 2017b), we exposed delicate skinks to two actual transport vectors: flights on a commercial airliner, and a 3 h drive in a car. As delicate skinks are a successful invasive species, and highly proficient at human‐assisted dispersal, we predicted that they would be highly tolerant of both types of transportation, and that their behaviour would not be impacted as a result of such transportation.

## 2 Methods

### 2.1 Lizard Collection and Housing

Ninety‐six adult male delicate skinks with full tails were collected from suburban Sydney (33°47′ S, 151°08′ E) by hand or mealworming (see Michelangeli, Wong, and Chapple 2016 for a detailed description of these collection methods). These collection methods have been shown not to bias the behaviours, or personality, of the delicate skinks collected (Michelangeli, Wong, and Chapple 2016). We only collected lizards with full tails, as tail loss can influence the subsequent behaviours of *Lampropholis* skinks (Downes and Shine 2001; Michelangeli et al. 2020). Similarly, as gravidity can influence the behaviour of skinks (Shine 2003), we only used adult males in our experiments. The skinks were collected from suburban Sydney, as it is one of the four known source regions for the introduction of the delicate skink to Lord Howe Island (Chapple, Miller, et al. 2013).

Skinks were transported by car back to animal housing facilities at Monash University. Upon arrival, skinks were housed in constant temperature rooms at approximately 22°C with 14 h light, 10 h dark cycle (see Michelangeli et al. 2016 for further housing details). Basking lamps were used in housing, providing a thermal gradient reaching 35°C, promoting natural thermoregulation. Skinks were fasted for at least 24 h prior to experimental trials because skinks are known to change their behaviour after large meals (Shine 2003).

### 2.2 Experimental Transportation Treatments

Experimental transportation treatments were conducted to determine the impact of both air transport, and car transport, on the behaviour of the delicate skink. For each type of transport, there was a treatment group that experienced the transportation, and a control group that was packaged in an identical manner, but did not experience any transportation. Each control and treatment group contained 24 delicate skinks. For the experiment, skinks were placed individually into a calico bag, and then placed into a compartmentalised box. For the duration of the experiments, iButtons (DS1923‐F5; Maxim Integrated Products, Sunnyvale, CA) were placed in packing boxes, measuring ambient temperature every minute.

#### 2.2.1 Car Transportation

Skinks in the experimental group were driven around suburban Melbourne (Australia) in an air‐conditioned car for a total of 3 h in April 2017, under a range of different driving conditions (e.g., freeway driving, suburban roads). The control skinks were kept packaged for the same length of time, but were kept in their temperature‐controlled room at Monash University. All post‐treatment behavioural trials (for both control and experimental animals) were completed within 5 h of the car transportation.

#### 2.2.2 Plane Transportation

Skinks in the experimental group were transported by a commercial pet transport company, Jetpets (www.jetpets.com.au) on the 12 April 2017. The skinks were transported from Monash University (Clayton campus) to the Melbourne airport by car (~1.5 h drive). They were then transported on two Qantaslink flights: (i) Melbourne to Mildura (QF2078, 8.30 am‐9.40 am), and (ii) Mildura to Melbourne (QF2018, 2.05 pm‐3.15 pm). Each flight was ~1 h 10 min in duration (~520 km), and the skinks remained packaged between the two flights. The aircraft used was a De Havilland (Bombardier) Dhc‐8 Series 300 (Dash‐8), which is the same type of plane used on flights between Sydney and Lord Howe Island, and the total flight time that delicate skinks were exposed to (~2 h 20 min) was equivalent to the 2 h 30 min flight time from Sydney to Lord Howe Island. Following the return flight, the skinks were driven by car from the Melbourne airport back to Monash University (~1.5 h drive). The control skinks were kept packaged for the same length of time, but were kept in their temperature‐controlled room at Monash University. All post‐treatment behavioural trials (for both control and experimental animals) were completed within 3 h of the experimental animals returning to Monash University.

### 2.3 Behavioural Trials

Each skink underwent a pre‐treatment trial (without prior confinement), 1 week prior to the experimental transportation, and a post‐treatment trial on the day of treatment. Behavioural trials were conducted as outlined in Chapple et al. (2011) and Michelangeli et al. (2016). Briefly, trials were conducted in an opaque test area (55 cm L × 32 cm W× 24 cm H) at approximately 22°C. Skinks were given 10 min to acclimate under a clear plastic container in the arena before the trial began. All trials were video recorded using JVC Everio GZ‐E100 video cameras. The behaviours were recorded during video playback using JWatcher (Blumstein et al. 2006). All experimental equipment was washed between trials using hot water and unscented detergent to prevent cross contamination of scents between trails.

#### 2.3.1 Activity

To measure activity, gridlines were drawn on the floor of the arena to divide it into 20 equal grid squares. During the experimental trials we recorded each time a skink moved into a new grid during the 45 min trial. The number of transitions between grid squares was taken as a measure of activity.

#### 2.3.2 Exploratory Behaviour

Exploratory behaviour was tested in the same test arena, but with the addition of an opaque partition, flush with the edge of the arena at the base, but with a 1.5 cm gap at between the arena edge and the partition at the top. To enter the second half of the arena, skinks must climb and squeeze past the barrier, replicating entering cargo. Over the 45 min trial, we recorded whether the lizard reached the other half of the arena, and if so, the time that it took to do so.

### 2.4 Statistical Analysis

Mean temperatures experienced during treatments were analysed between treatments and their respective controls using Wilcox tests. To investigate exploratory behaviour, survival analyses were used to determine the difference between time taken to cross the barrier before and after treatment for each group, using the survival package (Therneau and Grambsch 2000; Therneau 2024). Generalised linear mixed effects models using a *Poisson* distribution, and individual ID as a random effect, were used to determine if there was a significant difference in activity between trial stage and group. The lme4 package (Bates et al. 2015) was used. Given the initial activity of individuals was significantly different across some groups, to compare across different transport types, the difference in activity (post‐treatment—initial) was tested, with group as a predictor using an ANOVA (car package (Fox and Weisberg 2019)). For all analyses, R (Version 4.2.3) was used.

## 3 Results

**FIGURE 1:**
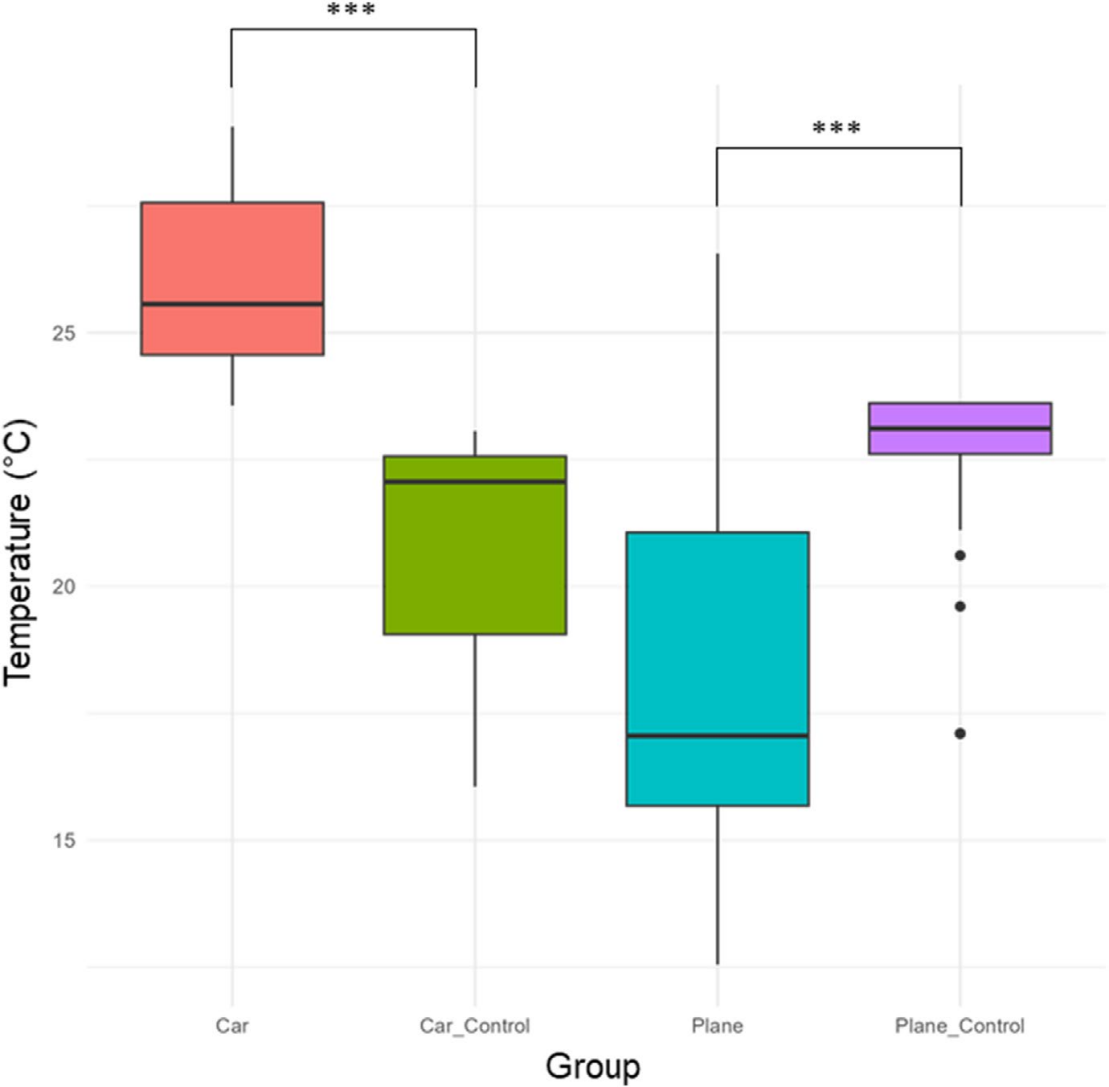
Boxplots showing mean and standard deviation of temperatures experienced during treatments. Statistical comparisons were made between treatments and their respective controls. *p* values are indicated as follows: *p < 0.05, **p < 0.01, ***p < 0.001.

Lizards experienced significantly different temperatures during the treatment and their respective controls; on average car temperatures were significantly hotter than the control (*W* = 65,536, *p*‐value < 0.0001), and plane temperatures significantly cooler than the control (*W* = 59,805, *p*‐value < 0.0001) (Figure 1). However, delicate skinks transported by plane experienced the greatest variation in temperature (Figure 1). The car transported lizards and the plane transported lizards experienced the warmest and coolest temperatures, respectively (Table 1 and Figure 1).

**TABLE 1:** Temperature extremes and averages (°C) experienced during respective experimental treatments.

Treatment	Average	Min	Max
Car transport	26.02	23.57	29.07
Car transport–control	21.14	16.06	23.06
Plane transport	18.38	12.55	26.56
Plane transport–control	23.08	17.10	23.61

**FIGURE 2:**
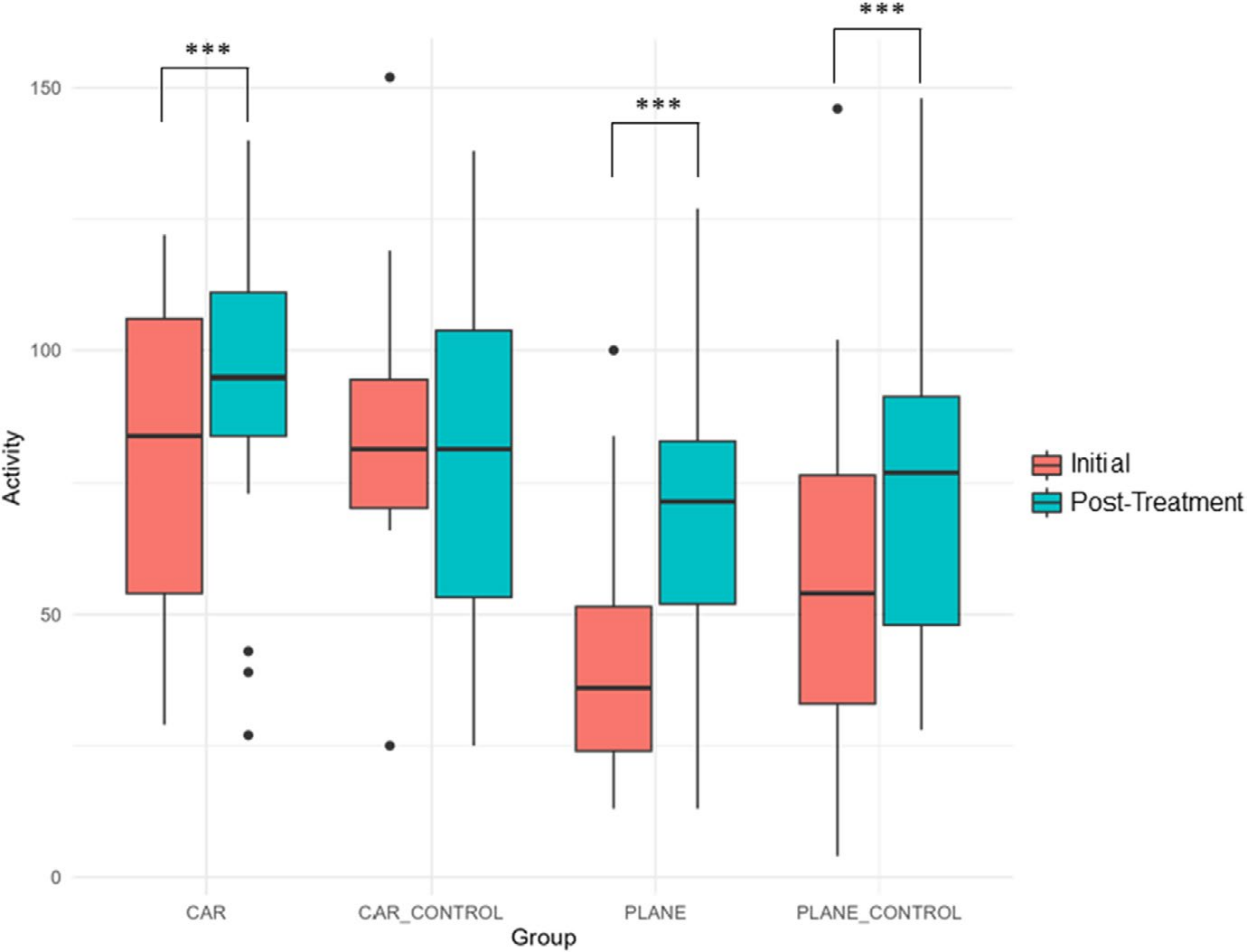
Boxplots showing mean and standard deviation of delicate skink activity. *p* values are indicated as follows: *p < 0.05, **p < 0.01, ***p < 0.001. See Table 4 for complete post hoc Tukey's comparisons.

**FIGURE 3:**
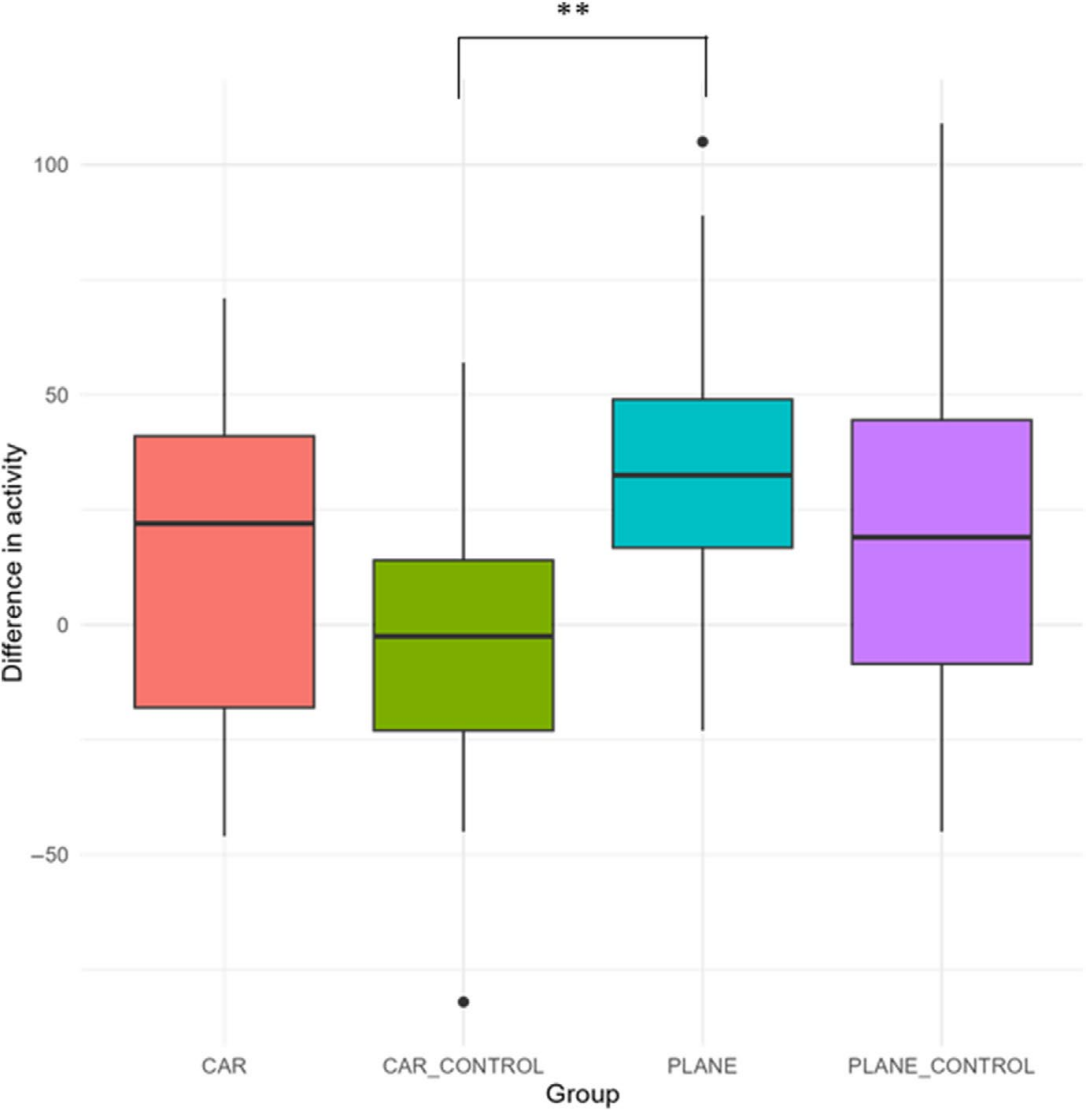
Boxplots showing mean and standard deviation of the difference in delicate skink activity in initial and post‐treatment trials. *p* values are indicated as follows: *p < 0.05, **p < 0.01, ***p < 0.001. See Table 5 for complete post hoc Tukey's comparisons.

Exploratory behaviour did not significantly differ between initial and post‐treatment trials for any control group or transport method (Table 2). However, for activity, trial, group, and their interactions were all found to be significant (Table 3). Post‐treatment, delicate skinks exhibited significantly higher activity in the car, plane, and plane control groups (Table 4, Figure 2). Group also had a significant impact on the change in activity levels between initial and post‐treatment trials (*F* value = 4.09, df = 3, *p* = 0.009), where there was a greater increase in activity in the plane compared to car control group (Figure 3, Table 5).

**TABLE 2:** Results of survival analyses comparing exploratory behaviour in initial and post‐treatment trials for each group, giving the log likelihood chi‐squared value (*X* ^2^), degrees of freedom (df) and *p*‐value.

Treatment	*Χ* ^2^	df	*p*
Group	5.00	3	0.17
Trial	2.55	1	0.11
Group × trial	2.57	3	0.46

**TABLE 3:** Results of mixed effects models using a Poisson distribution comparing activity in different trial stages or groups.

Treatment	Chi‐squared	df	*p*
(Intercept)	2414.95	1	**< 0.0001**
Trial	23.94	1	**< 0.0001**
Group	55.20	3	**< 0.0001**
Trial × group	152.15	3	**< 0.0001**

*Note:* Significant *p*‐values are indicated in bold.

**TABLE 4:** Results of post hoc Tukey's pairwise comparison tests for lizard activity levels as predicted by group and trial stage.

Contrast	Estimate	SE	df	*Z* Ratio	*p*
Initial car–post‐treatment car	−0.18	0.04	Inf	−4.89	**< 0.0001**
Initial car–initial car control	−0.07	0.13	Inf	−0.52	1.00
Initial car–post‐treatment car control	−0.01	0.12	Inf	−0.08	1.00
Initial car–initial plane	0.71	0.12	Inf	5.90	**< 0.0001**
Initial car–post‐treatment plane	0.11	0.12	Inf	0.95	0.98
Initial car–initial plane control	0.42	0.12	Inf	3.50	**0.01**
Initial car–post‐treatment plane control	0.10	0.12	Inf	0.86	0.99
Post‐treatment car–initial car control	0.11	0.13	Inf	0.86	0.99
Post‐treatment car–post‐treatment car control	0.16	0.13	Inf	1.30	0.90
Post‐treatment car–initial plane	0.86	0.12	Inf	7.37	**< 0.0001**
Post‐treatment car–post‐treatment plane	0.29	0.12	Inf	2.44	0.22
Post‐treatment car–initial plane control	0.60	0.12	Inf	4.97	**< 0.0001**
Post‐treatment car–post‐treatment plane control	0.28	0.12	Inf	2.33	0.28
Initial car control–post‐treatment car control	0.06	0.04	Inf	1.52	0.80
Initial car control–initial plane	0.77	0.12	Inf	6.36	**< 0.0001**
Initial car control–post‐treatment plane	0.18	0.12	Inf	1.49	0.81
Initial car control–initial plane control	0.49	0.12	Inf	4.00	**< 0.001**
Initial car control–post‐treatment plane control	0.17	0.12	Inf	1.40	0.86
Post‐treatment car control–initial plane	0.72	0.12	Inf	5.90	**< 0.0001**
Post‐treatment car control–post‐treatment plane	0.12	0.12	Inf	1.03	0.97
Post‐treatment car control–initial plane control	0.43	0.12	Inf	3.54	**< 0.001**
Post‐treatment car control–post‐treatment plane control	0.11	0.12	Inf	0.94	0.98
Initial plane–post‐treatment plane	−0.60	0.04	Inf	−14.86	**< 0.0001**
Initial plane–initial plane control	−0.29	0.12	Inf	−2.49	0.20
Initial plane–post‐treatment plane control	−0.61	0.12	Inf	−5.28	**< 0.0001**
Post‐treatment plan–initial plane control	0.31	0.11	Inf	2.72	0.12
Post‐treatment plane–post‐treatment plane control	−0.01	0.11	Inf	−0.09	1.00
Initial plane control–post‐treatment plane control	−0.32	0.04	Inf	−8.68	**< 0.0001**

*Note:* Significant *p*‐values are indicated in bold.

**TABLE 5:** Results of post hoc Tukey's pairwise comparison tests for difference in lizard activity between initial and post‐treatment trials as predicted by group.

Contrast	Estimate	SE	df	*t* Ratio	*p*
Car–car control	18.56	11.40	80	1.63	0.37
Car–plane	−18.59	10.60	80	−1.75	0.31
Car–plane control	−6.88	10.70	80	−0.64	0.92
Car control–plane	−37.15	10.80	80	−3.44	**< 0.001**
Car control–plane control	−25.44	10.90	80	−2.34	0.10
Plane–plane control	11.72	10.10	80	1.16	0.65

*Note:* Significant *p*‐values are indicated in bold.

## 4 Discussion

We demonstrate that human‐assisted dispersal only has a relatively minor impact on the behaviour of male delicate skinks. Transportation by plane did not influence the activity or exploratory behaviour of the delicate skink. However, although car transportation did not impact exploratory behaviour, it did result in an increase in activity (relative to the control group) in delicate skinks. Our study highlights that although the temperatures that transported lizards were subjected to during transit were more variable than those experienced by control lizards, the temperature extremes experienced remained well within the thermal tolerances for the species (e.g., Anderson et al. 2023). Below we discuss the implications of these findings for the invasion dynamics of the delicate skink.

### 4.1 Environmental Conditions During Air and Road Transportation

Whilst the transportation of animals is undoubtedly a stressful experience (Hartung 2003), our study indicates that delicate skinks were not exposed to extreme temperatures during either air or car transportation. Indeed, the temperatures that lizards experienced during transportation (car: 23.6°C–29.1°C, plane: 12.5°C–26.1°C) were well within the thermal tolerances for the species (CTmin: ~8°C, preferred temperature: ~26°C–27°C, CTmax: ~39°C; Anderson et al. 2023). As lizards in our study were packed into individual calico bags in a transport box, it is possible that they experienced more stable and ‘safer’ conditions than what stowaway individuals would actually be exposed to (e.g., White and Shine 2009). However, delicate skinks naturally seek shelter more than congeneric species (Chapple, Simmonds, and Wong 2011), which may act to buffer them from extreme environmental conditions during transit. In addition to temperature, another potential stressor during transportation is noise (Mancera et al. 2014). For instance, machinery noise has been shown to result in aversive behaviours and grouping tendencies in pigs (Stephens et al. 1985; Geverink et al. 1998), and aircraft noise triggers flight responses in desert ungulates (Weisenberger et al. 1996). The hearing range of lizards ranges from 1 to 3 kHz (Saunders et al. 2000), and with noise generated by vehicles generally below 2 kHz (Slabbekoorn and Peet 2003), it is within their hearing range of the delicate skink, and thus could prove a potential stressor. Such additional stressors could provide an explanation for why 8% (for stowaways from Australia into New Zealand) to 21% (for stowaways moving within New Zealand) of delicate skinks were dead when intercepted by New Zealand biosecurity agencies (Chapple, Whitaker, et al. 2013). In addition, temperatures experienced within cargo planes could potentially be more extreme than those that delicate skinks in our study experienced on the commercial airliner.

### 4.2 Human‐Assisted Transportation Has a Minimal Impact on the Behaviour of the Delicate Skink

Delicate skink behaviour (exploratory behaviour, activity) was not impacted by plane transportation, but individuals exposed to car transportation were found to be more active. However, individuals from both control groups were more active in the post‐transportation trials, which could represent a response to extended confinement. We are only aware of one comparable study to ours that replicated the conditions during human‐assisted dispersal (Petit, Greenlees, and Shine 2017a, 2017b). Petit, Greenlees, and Shine (2017a, 2017b) exposed cane toads ( *Rhinella marina* ) to car transportation for 2–4 h, and compared the impact of high versus low transport stress on the behaviour of individuals following translocation to a new site, or back to the original capture site. Behaviour of returned cane toads was unaffected, regardless of whether they experienced high or low transport stress (Petit, Greenlees, and Shine 2017a, 2017b); however, translocated toads had a greater dispersal distance and utilised different refuges (Petit, Greenlees, and Shine 2017a, 2017b). Thus, at least for the cane toad, the transportation process itself did not appear to impact the behaviour of the species, rather it was the exposure to a novel location which appeared to be stressful for the species. Future studies could therefore examine the response of delicate skinks being exposed to novel environments in order to gain a better understanding of why it is such a successful invasive species.

### 4.3 Conclusions

It has long been acknowledged that there is a dearth of knowledge regarding the initial stages of the introduction process, and particularly the initial transportation phase (Puth and Post 2005; Chapple, Simmonds, and Wong 2012). Our study provides important information on the capacity of an invasive species to cope with the stressors associated with human‐mediated transportation, and retain its normal behaviours upon arrival in a non‐native region. Obtaining such information for a broader range of invasive species will be important, as the ability of invaders to arrive in non‐native regions in good health and condition will be pivotal for their availability to successfully establish and spread out from the point of arrival (Blackburn et al. 2011; Chapple, Simmonds, and Wong 2012). Thus, such knowledge will improve our understanding of the factors that influence the success of biological invasions, and potentially for developing biosecurity strategies to prevent the successful establishment of new stowaways.

## Author Contributions

**Jaclyn Harris:** conceptualization (equal), data curation (equal), formal analysis (equal), investigation (equal), methodology (equal), visualization (equal), writing – original draft (equal). **Celine T. Goulet:** conceptualization (equal), data curation (equal), formal analysis (equal), investigation (equal), methodology (equal), supervision (equal), writing – review and editing (equal). **David G. Chapple:** conceptualization (equal), data curation (equal), funding acquisition (lead), methodology (equal), project administration (lead), resources (equal), supervision (equal), writing – original draft (equal).

## Ethics Statement

All animals were collected in accordance with the appropriate collection and research permits (New South Wales: SL101600, SL101425, Victoria: 10008294). All animal care and experimental procedures were approved by the Monash University Animal Ethics Committee (BSCI/2017/04).

## Conflicts of Interest

The authors declare no conflicts of interest.

## Supporting information


Data S1.

## Data Availability

Our data and R code are included in the Supporting Information.
